# Oxidation of Phenol by Hydrogen Peroxide Catalyzed by Metal-Containing Poly(amidoxime) Grafted Starch

**DOI:** 10.3390/molecules16129900

**Published:** 2011-11-29

**Authors:** Hany El-Hamshary, Mohamed H. El-Newehy, Salem S. Al-Deyab

**Affiliations:** 1 Petrochemical Research Chair, Department of Chemistry, College of Science, King Saud University, B.O. Box 2455, Riyadh 11451, Saudi Arabia; 2 Department of Chemistry, Faculty of Science, Tanta University, Tanta 31527, Egypt

**Keywords:** catalytic oxidation, phenol, hydrogen peroxide, chelating resin, polyamidoxime grafted starch

## Abstract

Polyamidoxime chelating resin was obtained from polyacrylonitrile (PAN) grafted starch. The nitrile groups of the starch-grafted polyacrylonitrile (St-*g*-PAN) were converted into amidoximes by reaction with hydroxylamine under basic conditions. The synthesized graft copolymer and polyamidoxime were characterized by FTIR, TGA and elemental microanalysis. Metal chelation of the polyamidoxime resin with iron, copper and zinc has been studied. The produced metal-polyamidoxime polymer complexes were used as catalysts for the oxidation of phenol using H_2_O_2_ as oxidizing agent. The oxidation of phenol depends on the central metal ion present in the polyamidoxime complex. Reuse of M-polyamidoxime catalyst/H_2_O_2_ system showed a slight decrease in catalytic activities for all M-polyamidoxime catalysts.

## 1. Introduction

Graft copolymerization of starch with vinyl monomers has gained importance in modifying the chemical and physical properties of starch. Hydrogels based on graft copolymerization of vinyl monomers and biopolymers like starch, are finding extensive use in various fields and technologies [1]. Among their valuable applications are their uses as efficient metal ion sorbents where their main chain units contain hydroxyl groups which can act as binding sites for metal ions [2,3]. Crosslinked poly(acrylonitrile) and amidoximated networks obtained from the post functionalization of acrylonitrile (AN) based polymers are also useful in metal ion pollution remediation [4,5,6]. Other applications of starch-graft polymers included phase transfer catalytic displacement and reduction [7], use as flocculating agents [8], oilfield and drilling mud additives [9]. Modified starch-*g*-polyacrylamide was found to be active in the catalytic decomposition of H_2_O_2_ at different pHs [10].

On the other hand removal of environmental pollutants such as phenol through oxidation processes is an approach that is attracting receives increasing attention [12]. Although methods of phenol removal that include sorption over natural surfaces like activated carbon [13], or sorption over organophilic clay that was modified with organic polymers [14,15] exist, is not always enough and the need to oxidize or reduce them to less harmful products is necessary. Oxidation of phenols using chemical reagents like hydrogen peroxide, permanganate, molecular oxygen and ozone, are widely used [16,17]. The use of hydrogen peroxide has the advantage of producing oxygen and can be used to augment biological degradation [18,19]. Besides, the adoption of H_2_O_2_ as an alternative of current industrial oxidation processes offer environmental advantages, some of which are: (a) replacement of stoichiometric metal oxidants; (b) replacement of halogens; (c) replacement or reduction of solvent usage; and (d) avoidance of salt by-products. Hydrogen peroxide works either alone or with a catalyst. Iron is the most common homogeneous catalyst for hydrogen peroxide [20]. Heterogeneous catalysts involve metal oxides [21] and supported metal oxides [22], and polymeric supports [23,24].

However, post-chemical modifications on starch-grafted polymers and applications in chemical reactions as catalyst support or as carriers in separations and organic synthesis are still lacking. The purpose of this investigation is to explore a new catalyst-support based on a natural renewable resource and to apply it for phenolic wastewater treatment by a catalytic peroxide oxidation process. Starch is a natural plant product and it has been modified by acrylonitrile and further converted into an amidoxime form for use as catalyst support with a minimum amount of auxiliary chemicals and solvents for the realization of some of the fundamental objectives of green chemistry.

## 2. Results and Discussion

### 2.1. Grafted Copolymer St-g-PAN

Starch graft copolymers can be prepared by generating free radicals on the starch backbone and then allowing these macroradicals to react with monomer. Although a number of methods are available for initiating the grafting reactions of vinyl monomers onto starch substrates, the ceric-ion-induced redox initiation method has been preferred over the years [25,26]. This is because when starch is oxidized by ceric ions, the free radicals capable of initiating vinyl polymerization are usually formed exclusively on the starch backbone by a single electron transfer, thus minimizing the formation of homopolymer and increasing the grafting efficiency.

**Figure 1:**
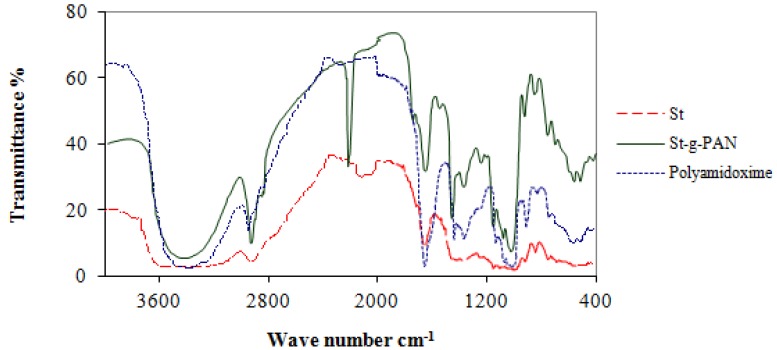
FTIR spectrum of starch, St-*g*-PAN and polyamidoxime polymers.

Polyacrylonitrile grafted starch was thus obtained from the reaction of starch and acrylonitrile using ceric ammonium nitrate as free radical initiator (0.02 M) and sulfuric acid (0.045 M), respectively. The reaction temperature was maintained at 45 °C for 180 min. The percentage of graft efficiency was 66.6%. Figure 1 shows the FTIR spectra of the initial substrate starch and the St-*g*-PAN. The spectrum shows absorptions at 2,245 cm^−1^ due to CN stretching, and at 3,370 cm^−1^ due to OH stretching of starch. 

### 2.2. Synthesis of Starch-Based Poly(amidoxime) Resin

**Scheme 1:**
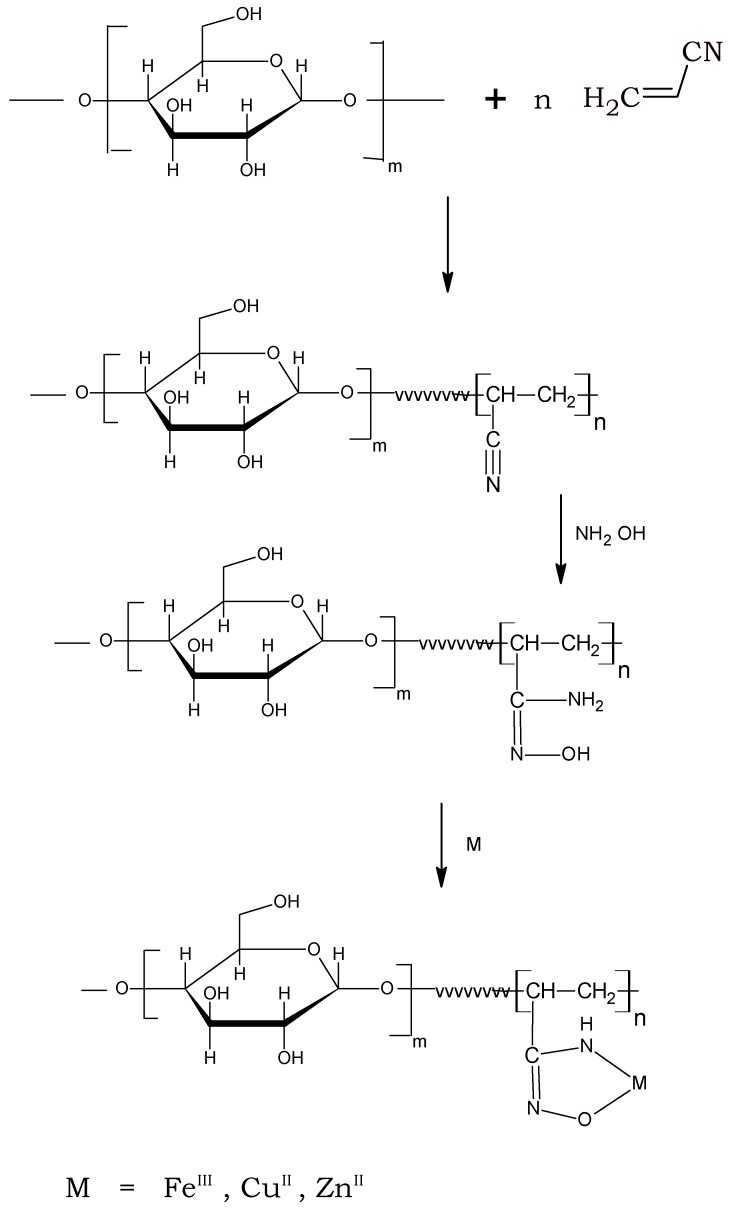
Synthetic route to starch-g-polyamidoxime.

The cyanide functional groups of the graft copolymer St-*g*-PAN were reacted with hydroxylamine in an alkaline medium to yield amidoxime groups. The ratio of hydroxylamine was in excess to St-*g*-PAN, the reaction was done for 2 h and temperature was 70 °C. A mixture of methanol-water ratio 5:1 was used as reaction medium. The synthetic procedure is shown in Scheme 1. Upon amidoximation, the nitrogen content increases by 1.48% in comparison to the precursor, thus supporting the functionalization by post reaction. Similar results were observed before upon amidoximation acrylonitrile-grafted carboxymethylated starch [27,28]. The formation of the amidoxime group was confirmed qualitatively by treatment with iron (III) chloride to give a purplish-red colored complex.

FTIR spectra confirm that the nitrile groups are converted to amidoxime groups under the previously described experimental conditions. Figure 1 shows the IR spectra of starch where bands appear at 3,370 cm^−1^ and at 1,654 cm^−1^ are due to OH stretching. Other characteristic bands were observed at 2,930 cm^−1^ and 1,022 cm^−1^ due to CH stretching and bending, respectively. The IR spectrum of purified St-*g*-PAN is also shown in Figure 1. The characteristic sharp absorption band of CN group appears at 2,245 cm^−1^ (CN stretching) in addition to the broad band at 3,370 cm^−1^ for the OH group. The presence of nitrile band in the IR spectrum indicates that PAN has successfully grafted onto the starch. After treatment of St-*g*-PAN with hydroxylamine the sharp band of the CN group disappeared (Figure 1) due to formation of amidoxime group (C=N) with a band appearing at 1,651 cm^−1^, and the amide II band of N-H at 1,570 cm^−1^. The peak at 938 cm^−1^ may be assigned to the N–O bond of the oxime group [28].

The polymeric catalyst was prepared by mixing the grafted polyamidoxime material with metal nitrate solutions overnight with stirring. After separation and purification, the amount of the metal bound to the grafted polyamidoxime material was determined by the reaction of the polyamidoxime M-complex with standard HCl solution and the amount of metal bound was determined by titration of the liberated metal using standard EDTA solution (Table 1). The results showed that binding of the metal ion or their affinity to polyamidoxime resin was in the order: Cu^2+^ > Fe^3+^ > Zn^2+^. A similar trend of metal complexation was observed before [6].

**Table 1:** Amount of metal loaded on the graft copolymer.

Metal/g	Cu mmol/g	Fe mmol/g	Zn mmol/g
Polyamidoxime	10	6.5	6.2

### 2.3. Thermogravimetric Analysis

**Figure 2:**
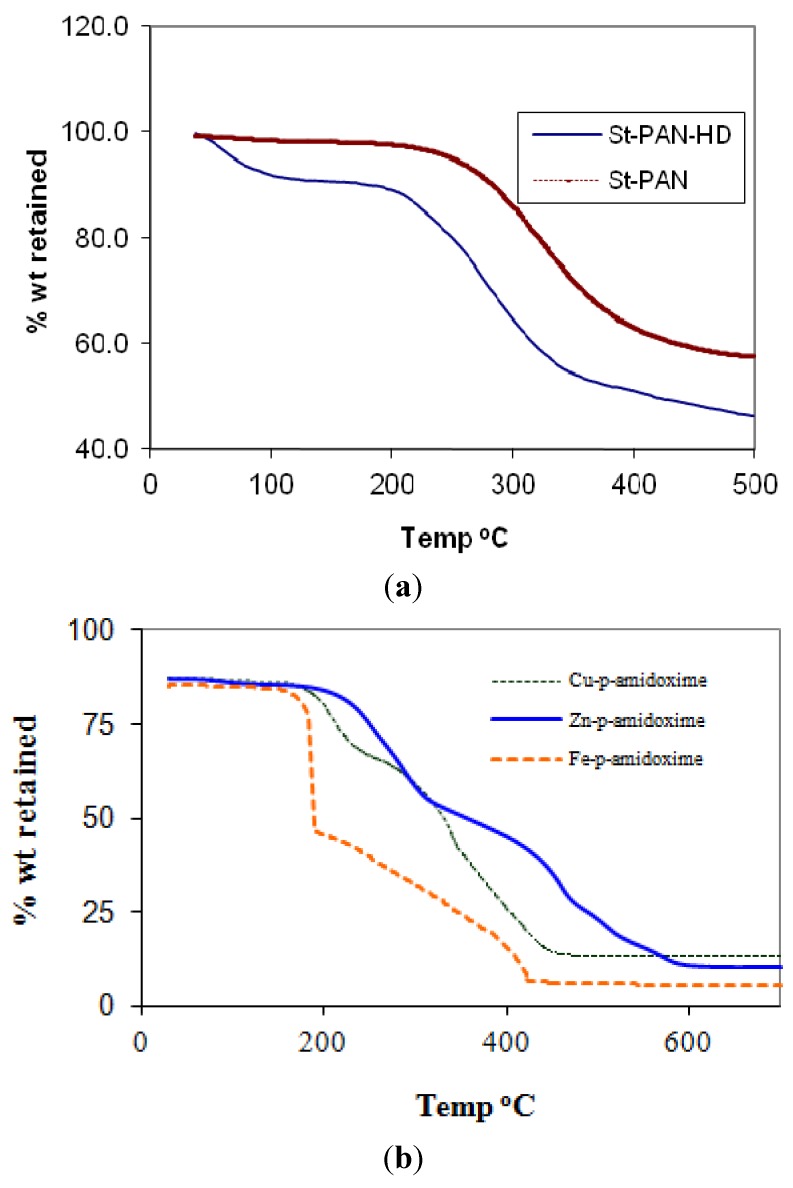
(**a**) Thermo gravimetric analysis for St-*g*-PAN; (**b**) Thermo gravimetric analysis for polyamidoxime metal-complexes.

The thermal degradation of poly(amidoxime) resin and polyacrylonitrile (PAN) grafted starch was performed with a heating rate of 10 °C/min in an N_2_ atmosphere and the TG curves are presented in Figure 2a. The main weight loss of PAN grafted starch occurs between the range of 235–380 °C while for poly(amidoxime) resin it occurs in the range 112–360 °C. The poly(amidoxime) resin was less stable than PAN grafted-starch and started to degrade at 70 °C up to a temperature of 345 °C. In the final stage, about 42% weight remains in PAN grafted sago starch, whereas 53% weight remaining in poly(amidoxime) resin was observed up to 500 °C. This difference is obviously due to the modification and amidoximation processes.

The polyamidoxime-metal complexes were subjected to thermogravimetric analysis to study their thermal stabilities. A clear difference in the thermal behavior between the polyamidoxime-M-complexes was observed. Figure 2b shows a characteristic three-step thermogram for polyamidoxime-Fe complexes where a sharp weight loss is observed in the first stage at 154 °C. A rapid decomposition occurs in the following stages at 201 and 431 °C with a major weight loss of 59.4%. The maximum temperature of decomposition occurs at 500 °C. When polyamidoxime polymer was loaded with Cu its thermal behavior was enhanced compared to the Fe-polymer complex. Zn-polyamidoxime looks to be more stable than the Fe-polyamidoxime polymer and Cu-polyamidoxime polymers, but the char yield was slightly less than that of the Cu polymer and higher than that of the Fe polymer complex. Amidoximation and metallation however, seem to render the base polymer more susceptible to thermal degradation. Thus from the TGA analysis the changes in the observed thermograms due to metallation of the polymer with the different metals can therefore be attributed.

### 2.4. Oxidation of Phenol

In order to evaluate the catalytic activity of the polyamidoxime-metal complexes, the catalytic oxidation of phenol using H_2_O_2_ as oxidant was studied to prove the efficiency of the catalysts. The ratio of H_2_O_2_ to phenol was 10:1. Reactions were carried out for a fixed period of time (4 h at 25 °C under air), and the change of the phenol concentration was followed from the absorbance at 410 nm using spectrophotometer. The percentage of phenol conversion, were calculated from the difference in initial concentration.

Different metals have special oxygen transfer properties which improve the utility of hydrogen peroxide through generating the highly reactive hydroxyl radicals (**^•^**OH). The conversion of phenol contributed to the decomposition of hydrogen peroxide, which generated ^•^OH free radical. The results summarized in Table 2 indicate that conversion of phenol was higher with Cu-polyamidoxime catalyst than with Zn-polyamidoxime and Fe-polyamidoxime catalysts, although iron is the most common metal used for treatments of industrial organic wastes and municipal waste waters [18].

**Table 2:** Effect of amount and catalyst efficiency.

Catalyst	Catalyst weight	% phenol conversion
Fe-Polyamidoxime	0.05	85
	0.10	87
	0.20	88
Cu-Polyamidoxime	0.05	86
	0.10	92
	0.20	95
Zn-Polyamidoxime	0.05	83
	0.10	85
	0.20	86

All reactions were carried out for 4 h at 25 °C and pH 9, phenol concentration was 1 × 10^−3^ M, and H_2_O_2_ was 0.01 M. The H_2_O_2_/phenol molar ratio is 10/1. Total volume of reaction mixture was maintained at 25 mL.

However, the conversion of phenol using Fe^III^ catalyst system was less than in case of Cu and Zn (Table 2). This could be due to formation of colloidal Fe^III^ which may catalytically decompose H_2_O_2_ into oxygen and water without forming hydroxyl radicals. On the other hand, when the amount of catalyst increased from 0.05 to 0.2 g, the percentage of phenol conversion increased with increasing amount of catalyst dosage in the solution. The enhancement with the addition of catalyst could be due increased number of catalytic sites for reaction with phenol molecules. The presence of more catalytic active sites is proposed to the increase in efficiency of the decomposition rate of hydrogen peroxide. Therefore, high conversion for oxidation of phenol with increasing the catalyst dosage can be expected based on the results of this work. When the reaction was carried out without the metal-polyamidoxime catalysts no detectable conversion of phenol was observed.

### 2.5. Effect of pH

Fenton type reactions are dependent on pH [19] during oxidation reactions. The effective pH range was usually in between 3–6 [19], because the formation of free radicals is enhanced and thus facilitates oxidation of organic materials. Thus, it is expected in the present work that the phenol conversion is dependent on the **^•^**OH formation produced from decomposition of H_2_O_2_ in the M-polyamidoxime catalyst/H_2_O_2_ system. All M-polyamidoxime catalysts showed maximum activity at pH 7 (Table 3). Although this pH is higher than other previous results, this could be due to high *p*K_a_ value of phenol (10.0).

**Table 3:** Effect of pH.

Catalyst	pH	% phenol conversion
Fe-Polyamidoxime	9	85
	7	91
	5	47
Cu-Polyamidoxime	9	85
	7	94
	5	54
Zn-Polyamidoxime	9	83
	7	90
	5	44

All reactions were carried out for 4 h at 25 °C and the weight of catalyst was 0.056 g, phenol concentration was 1 × 10^−3^ M, and H_2_O_2_ was 0.01 M. The H_2_O_2_/phenol molar ratio is 10/1. Total volume of reaction mixture was maintained at 25 mL.

The possible reason is that the decrease in acidity of M-polyamidoxime catalytic oxidation benefited the formation rate of **^•^**OH free radical and enhanced the decomposition of organic compounds. It can be concluded that the initial pH in solution dominated the phenol conversion and oxidation rate of organic materials.

### 2.6. Influence of Reaction Temperature

Phenol oxidation was carried out with M-polyamidoxime catalyst/H_2_O_2_ system at temperatures of 25, 50 and 65 °C. For all catalyst systems the percentage of phenol conversion showed a pronounced increase with increasing temperature at 50 and then decreased at 65, as presented in Table 4. The higher reaction temperature could enhance the thermal degradation rate of H_2_O_2_. The free radical concentration usually increases with increasing reaction temperature in such systems. Similar results were previously observed for catalytic wet phenol oxidation CWPO [29,30].

**Table 4:** Effect of temperature.

Catalyst	Temperature °C	% Phenol conversion (pH 9)
Fe-Polyamidoxime	25	85
	50	90
	65	74
Cu-Polyamidoxime	25	86
	50	98
	65	81
Zn-Polyamidoxime	25	83
	50	93
	65	77

All reactions were carried out for 4 h with 0.056 g of catalyst. Phenol concentration was 1 × 10^−3^ M, and H_2_O_2_ was 0.01 M. The H_2_O_2_/phenol molar ratio is 10/1. Total volume of reaction mixture was maintained at 25 mL.

### 2.7. Catalyst Reuse

Recycling of M-polyamidoxime catalyst/H_2_O_2_ system showed a slight decrease in catalytic activities for all metal-polyamidoxime catalysts in the first cycle and greater decreases in later application. Such decreases in catalytic activity could be due to accumulation of reaction products in the polyamidoxime resin near the active site. Another reason for catalyst deactivation may be due to the possibility of active metals leaching from the solid materials.

## 3. Experimental

Phenol and acrylonitrile (Aldrich) were used as received. Starch was supplied by BDH Chemicals. The nitrate salts of Fe^III^, Cu^II^ and Zn^II^ used for ligand metallation were of reagent grade and used as received. Hydrogen peroxide (Riedel de Haën, 30%) was used as the oxidant in solution. Ceric ammonium nitrate (BDH, Poole, England), hydroxylamine hydrochloride (Fluka, Messerschittstr, Switzerland), methanol (Baker, NJ, USA), and other chemicals used were analytical reagent grade. Briton-Robinson buffer solutions pH 5, 7 and 9 were used in the study. Glacial acetic acid (2.3 mL), phosphoric acid (2.7 mL) and boric acid (2.472 g) were dissolved to 1.0 L. Then 50 mL portions of this solution were taken and pH was adjusted to 5–9 by addition of appropriate amount of 1.0 M NaOH.

Elemental microanalysis was done at the Central Laboratory of Microanalysis at Cairo University. IR spectra were recorded on a Perkin Elmer 1430 ratio recording IR spectrophotometer from KBr pellets. The thermogravimetric analysis was carried out on a Perkin Elmer Pyris Diamond instrument from 25 to 500 °C.

### 3.1. Preparation of Graft-Copolymers

Starch (4.0 g) and distilled water (200 mL) were placed into a 1-L ﬂask, which was equipped with a mechanical stirrer and condenser, and immersed in a thermostated water bath. The starch slurry was preheated at 80 °C for about 30 min. It was continuously stirred and purged with N_2_ gas. After gelatinization, the ﬂask contents were then cooled to 45 °C and sulfuric acid (2 *N*, 4.5 mL) was added to the mixture. After 5 min, a 0.1 M ceric ammonium nitrate solution (20 mL) was added to the mixture. Ten min later, acrylonitrile monomer (9.6 mL) was added. The reaction mixture was stirred for 1.5 h. After a complete reaction, the product was cooled and poured into methanol (500 mL) to induce precipitation. The grafted copolymer was washed several times with methanol-water (4:1), and then the product was dried at 50 °C to a constant weight (10.4 g). This grafted copolymer was used in the amidoxime preparation. Elemental analysis gave: C 50.04 (calcd. 50.47), H 5.03 (calcd 5.601), N 6.66 (calcd 6.54). FTIR spectra (KBr) showed absorptions at 2,245 cm^−1^ (CN stretching), and at 3,370 cm^−1^ (OH stretching).

#### 3.1.1. Conversion of Graft-Copolymer into Polyamidoxime

The conversion of the nitrile groups to amidoxime groups was carried out by the treatment of St-*g*-PAN with hydroxylamine hydrochloride (NH_2_OH·HCl) in an alkaline medium. First a solution of neutral hydroxylamine was made by dissolving hydroxylamine hydrochloride (10 g) in aqueous methanol solution (methanol-water 5:1, 80 mL). Then, the HCl of NH_2_OH was neutralized by NaOH solution and the precipitate of NaCl was removed by filtration. The pH of the reaction solution was adjusted to pH 10 by adding NaOH solution. The reaction medium was kept at a methanol to water ratio of 5:1. Then grafted St-*g*-PAN copolymer (4.0 g) was placed into the two-neck flask, which was equipped with a mechanical stirrer, condenser and thermostat water bath. Then the above-prepared hydroxylamine solution was added to the flask, and the reaction was carried out at 70 °C and 2 h duration. After completion of the reaction, the resin was separated from solution by filtration and washed several times with aqueous methanol (methanol-water 4:1). Finally, the resin was filtered off and then dried under vacuum at 40 °C to a constant weight. Elemental analysis gave: C 41.27 (calcd 43.47), H 5.18 (calcd 5.601), N 16.88 (calcd 11.34). The conversion of nitrile group was qualitatively examined by shaking a wet sample (0.1 g) with iron (III) chloride whereby a purplish-red colored complex was observed.

### 3.2. Synthesis of Polymeric Catalyst

The polymeric catalyst was prepared by mixing the grafted polyamidoxime material (1.0 g) with metal nitrate solution: Fe^III^, Cu^II^ and Zn^II^ (30 mL, 0.1 M) for 24 h. The grafted copolymer complex was filtered and washed with deionized water. The amount of the metal bound to the grafted material was determined by reacting the polymer complex with standard HCl solution and the amount of metal bound was determined by titration of the liberated metal using standard EDTA solution (Titrisol, Merck) (Table 1). 

### 3.3. Catalytic Oxidation of Phenol

Oxidation experiments were conducted in a 50-mL glass reactor and stirred with a magnetic stirrer. In a typical experiment: the polymeric catalyst previously prepared was added to H_2_O (10 mL) placed in the glass reactor. The pH was adjusted to 9.0 by addition of buffer solution (5 mL). The mixture was stirred magnetically for 5 min and a solution of phenol (1 × 10^−3^ M, 2.5 mL) was then added. The reaction flask was placed in a thermostated water bath for 10 min. The reaction was started by injecting H_2_O_2_ (from 0.5 M aqueous solution, 0.5 mL) whereby the total reaction volume was maintained at 25.0 mL. The concentration of H_2_O_2_ in reaction mixture is therefore 0.01 M. This makes the ratio of H_2_O_2_ to phenol 10:1. The reactions were run for 4 h at 25 °C under air. At the end of reaction, the solid polymer was filtered off, and washed with methylene chloride. A blank catalytic oxidation experiment was carried out in absence of polymeric catalyst.

### 3.4. Catalyst Re-Use

Reuse of the M-polyamidoxime catalyst/H_2_O_2_ system was tested as follows: oxidation of phenol was carried out under the standard reaction conditions of Table 2. After the completion of the reaction, the M-polyamidoxime catalyst was filtered off and washed with methylene chloride and dried well. The dried catalyst was placed in the reaction flask and initial amount of phenol was again fed into reaction mixture. Data are listed in Table 5.

**Table 5:** Catalyst reuse ^a^.

Catalyst ^a^	% Phenol conversion
Fe-Polyamidoxime	71
Cu-Polyamidoxime	80
Zn-Polyamidoxime	77

^a^ All reactions were carried out for 4 h at 25 °C and pH 9 and weight of catalyst was 0.056 g. Phenol concentration was 1 × 10^−3^ M, and H_2_O_2_ was 0.5 M. Total volume of reaction mixture was maintained at 25 mL.

## 4. Conclusions

Polyamidoxime chelating resin was obtained from polyacrylonitrile (PAN) grafted starch. Fe^III^, Cu^II^ and Zn^II^ were supported onto polyamidoxime grafted starch and used to catalyze the oxidation of phenol by H_2_O_2_. The oxidation of phenol depends on the central metal ion present in the polyamidoxime complex. Reuse of M-polyamidoxime catalyst/H_2_O_2_ system showed a slight reduction in catalytic activities for all M-polyamidoxime catalysts. The results described in this investigation indicate that these metal complexes of polyamidoxime grafted starch can serve as good catalyst systems for phenol oxidation and the treatment of related organic pollutants in industrial effluents and wastewater.
